# Optical coherence tomography angiography of the macula of high myopia in children and adolescents

**DOI:** 10.1186/s40942-024-00532-w

**Published:** 2024-02-05

**Authors:** Marwa Mahmoud Abdellah, Ahmed Ali Amer, Zeiad Hasan Eldaly, Mohamed Abdellatif Anber

**Affiliations:** 1 Ophthalmology Department, Sohag Faculty of Medicine, Sohag University, Sohag, 82525 Egypt; 2 Ophthalmology Department, Qena Faculty of Medicine, South Valley University, Qena, Egypt; 3 Ophthalmology Department, Assiut Faculty of Medicine, Assiut University, Assiut, Egypt

**Keywords:** Central macular thickness, Foveal avascular zone, High myopia, High myopic adolescents, Highly myopic children, Macular vessel density in myopes, Myopic choroidal neovascularization, Myopic maculopathy, Optical coherence tomography angiography

## Abstract

**Background:**

High myopia represents a health issue and leads to the development of complications that threaten vision. The study of macular changes in high myopia patients has undergone great advances with updated technology via new spectral optical coherence tomography (OCT) and Optical coherence tomography angiography (OCTA). Most of related studies have focused on the adults and additional studies need to investigate macular changes in children and adolescents. This study aimed to evaluate the changes in the macular structure by OCT and the macular vessel density in high myopia in children and adolescents by OCTA.

**Methods:**

A cross-sectional comparative study. The population was divided into two groups: group 1 (4–11 years) and group 2 (12–18 years). The results were comparable to those of control study of the same age group. The two high myopia groups and the control groups were examined by macular OCT and OCTA to evaluate macular thickness and vessel density in the superficial and deep capillary plexuses.

**Results:**

OCT measurements of patients in group 1 revealed that central macular thickness was significantly lower in high myopia group than in the control group and measured 220.91 ± 27.87 μm and 258.23 ± 17.26 μm, respectively, (*P* < 0.0001). However, in group 2 the central macular thickness in the high myopia group and control group was 236.32 ± 27.76 μm and 247.09 ± 16.81 μm respectively, and the difference was not statistically significant (*P* = 0.09). The parafoveal macular thickness and the perifoveal macular quadrants thickness were significantly lower in high myopic children and high myopic adolescents (*P* < 0.0001) than age matched controls. The parafoveal and perifoveal vessel densities in the superficial and deep capillary plexuses were lower in the high myopia groups than in the age-matched controls in both groups with a few segment exceptions in group 1. The FAZ was significantly wider in group 1 than in the age-matched emmetropes (*P* = 0.02). The FAZ was wider in group 2 than controls, but the difference was not statistically significant, (*P* = 0.75).

**Conclusion:**

High myopic children and adolescents have thinner macular thickness than comparable age-matched emmetropes and have less vessel density in superficial and deep capillary plexuses with a wider FAZ.

**Supplementary Information:**

The online version contains supplementary material available at 10.1186/s40942-024-00532-w.

## Background

High myopia represents a health issue and leads to the development of complications that threaten vision, including retinal detachment, and myopic macular degeneration (MMD), in addition to complicated cataracts [1, 2].

High myopia prevails in 0.6–4.9% of children of different races [3]. In recent years, myopia has clearly presented at younger ages than before [4].

The prevalence of high myopia has significantly increased in recent decades worldwide, with a great impact on the health services provided to manage and prevent ocular complications related to high myopia [5].

The importance of studying high myopia as a single entity comes from the retinal complications of high myopia, as myopic macular degeneration can result in significant permanent visual impairment [6].

Myopia imaging is highly important for early case detection, precise diagnosis, prognosis prediction, planning, and management [7].

The study of macular changes in patients with high myopia has advanced substantially with updated technology such as new spectral optical coherence tomography (OCT), which is commonly employed for the assessment of the retina and optic nerve by providing the qualitative and quantitative evaluation of macula and retinal nerve fiber layer (RNFL) [8].

In the past, pathological changes in the retinae of eyes with high myopia were difficult to investigate, but only in enucleated eyes, by histological examination [9]. However, OCT can be used to diagnose structural changes, such as chorioretinal atrophy, myopic neovascularization, dome-shaped macula, macular hole, myopic traction maculopathy, and macular schisis.

Few studies have investigated fundus changes in children with high myopia. For instance, Kobayashi et al. carried out a review of the fundus features among children with high myopia. According to this review, mild peripapillary chorioretinal atrophy was observed in 16.3% of the examined eyes. They reported that no patients demonstrated geographic atrophy signs and postulated that aging, accompanied progressive mechanical stretching, could play a significant role in developing myopic chorioretinal degeneration [10].

Most related studies have examined high myopia in adulthood. However, data about the onset of retinal or choroidal changes are missing, and whether these changes started in early childhood has not been discussed. However, Yokoi reported that peripapillary diffuse chorioretinal atrophy in childhood is considered to be a sign of pathological myopia in adulthood [11].

Since 1977, researchers have spotted changes in the morphology of the vasculature system among patients with myopia [12]. However, investigating retinal vasculature has been challenging because of the many issues associated with imaging modalities. For instance, fluorescein angiography (FA) is an invasive procedure, that is not able to provide quantified data [13] and is difficult to perform in early childhood.

In the last decade, developments in OCT angiography (OCTA) have given us the opportunity to study blood flow and vascular layers rapidly and noninvasively [14], these advancements can help to detect vascular-related myopia- macular lesions, including myopic choroidal neovascularization (m CNV), early in life, and can be used to examine children in a friendly way.

Therefore, this study aimed to evaluate the changes in macular structure by OCT and macular vessel density in children and adolescents with high myopia by OCTA and to evaluate whether retinal examination is needed in high myopic children.

## Patients and methods

This study involved cross-sectional comparative research. All procedures performed in studies involving human participants were in accordance with the ethical standards of the Medical Research Ethical Committee (MREC) and with the 1964 Helsinki Declaration and its later amendments or comparable ethical standards. Ethical approval for the study was obtained from Sohag University, Sohag Faculty of Medicine under IRB registration number Soh-Med-23-3-14PD.

Informed written consent was obtained from all individual participants and from their guardians (younger 16 years) included in the study.

This study aimed to examine macular OCT and OCTA findings in high myopia children and adolescents aged 4 years to 18 years.

The cases were divided into two groups: group 1 (4–11 years) and group 2 (12–18 years), to the determine the age at which stage the OCT and OCTA findings could change. The results were comparable to those of the control study of the same age group.

Thirty-four eyes with high myopia in thirty-four children (4–11 years) and thirty-four eyes with high myopia in adolescents (12–18 years) were included.

The inclusion criteria for the myopic participants were as follows: axial myopia ≤ -6 diopters, axial length > 26 mm, no corneal diseases, no glaucoma, no history of trauma, no systemic diseases, and no previous ocular surgeries. The inclusion criteria for the control group were refraction around the emmetropia (between SE + 1.00 and − 1.00), no corneal diseases, normal IOP, no posterior segment diseases, no history of trauma or intraocular surgery, no history of prematurity, and no associated neurological diseases. The controls were chosen from among the normal children who presented to the outpatient clinic with simple conjunctivitis, or from among the volunteers of patients’ relatives.

All cases (myopic and control) underwent anterior segment examination and IOP measurement by Tonopen, and axial length and keratometry were measured by an IOL master (500; Carl Zeiss Meditec, Jena, Germany); manifested refraction, cycloplegic refraction by 0.5% cyclopentolate hydrochloride, and best corrected visual acuity (BCVA) were recorded. Optical coherence tomography and optical coherence tomography angiography to evaluate the macula. Examining the participants was carried out using the XR-Avanti OCT device (RTVue-XR Avanti; Optovue, Inc., Fremont, California, USA), with the 840 nm wavelength laser to capture 70,000 A-scans/second; 304 A-scans made up a B-scan, whereas 304 vertical and horizontal lines were sampled in the scanning area to obtain 3D data cube 3 × 3 volume scans centered on the fovea.

Measuring the macular region’s retinal thickness was carried out via the macula map protocol 512 A-scans × 128 B-scans. The measurement of central macular thickness was carried out from the boundary from the internal limiting membrane (ILM) to the outer boundary of the retinal pigment epithelium (RPE).

The acquisition and processing of the OCTA image. In the OCTA system (RTVue-XR Avanti; Optovue, Inc., Fremont, California, USA), Angio Vue used SSADA to identify vessels with blood flow according to the intrinsic motion contrast offered by the flowing erythrocytes, allowing the possibility of noninvasively obtaining 3D maps of the retina and choroidal microvasculature.

During scanning, two B-scans were taken at every fixed location, whereas two orthogonal OCTA volume scans (horizontal and vertical) were utilized to reduce fixation changes and motion artifacts. Evaluating the macular vessels required the acquisition of a 6 mm×6 mm scanning area centered on the macula. This area was automatically split into two segments, namely the superficial and deep capillary plexuses. The superficial capillary plexus en face image was divided into segments with an inner boundary 3 μm beneath the internal limiting membrane (ILM) and an outer boundary 15 μm beneath the inner plexiform layer (IPL). In contrast, the deep capillary plexus boundaries were defined as 15 to 70 μm beneath the IPL. The superficial and deep vascular density were automatically measured as the percentage of the region measured with flowing blood vessels by the OCT machine’s inner software (version 2017.1.0.155) (see additional file 1).

Two experienced examiners independently examined and assessed the participants. We excluded poor quality images with a signal strength index < 40 or registered image sets with residual motion artifacts. Multiple measurements were carried out. Only the best-centered measurement with good signal strength was selected for analysis. Measurements of the macular analysis included central macular thickness (central 1 mm disc) and macular thickness in two concentric circles: 3 mm (parafoveal circle) and 6 mm (perifoveal circle) in diameters centered at the fovea (see additional file 2).

The measurements in this study were adjusted using modified Littmann’s formula who described by Bennet et al. who used a magnification factor of the eye to make this correction [15], expressed as: t = p · q · s, where t is the actual fundus measurement, s is the OCT measurement, p is the magnification factor for the imaging system camera, and q is the magnification factor related to the eye [16].

The parafoveal and perifoveal circles were divided into four quadrants: temporal, inferior, nasal, and superior based on the nine regions A1-9 following the Early Treatment Diabetic Retinopathy Study (ETDRS Research Group 1985).

The foveal avascular zone (FAZ) represents an avascular area resulting from the lack of terminal capillaries in the deep and superficial plexuses [17] and was measured automatically by the device software (see additional file 3). FAZ measurements were corrected according to MATLAB script (MathWorks, Natick, MA) previously described by Linderman [18] to avoid the effect of ocular magnification affected by axial length. The area of the corrected FAZ was calculated as follows: A corrected = A nominal (ALs/Alm)2, where Als is the axial length of the subject in mm), and Alm is the axial length assumed for the model eye (23.95 mm).

The exclusion criteria for OCT angiography scans were as follows: [1] a signal strength index < 40 [2]. Low-quality images with severe artifacts due to poor fixation; motion artifacts.

### Statistical analysis

Data were analyzed using STATA version 17.0 (Stata Statistical Software: Release 17.0 College Station, TX: StataCorp LP.). The Shapiro–Wilk normality test was used to determine the distribution of different variables. Quantitative data were represented as mean and standard deviation. Data were analyzed using student using Mann-Whitney test was as the data were not normally distributed. Qualitative data are presented as numbers and percentages and were compared using Chi square test. P value was considered significant if it was less than 0.05.

## Results

The mean axial length was 27.82 ± 1.30 mm in the high myopia group 1, while the axial length 2 was 27.97 ± 0.83 mm in group 2. The mean manifest refraction in group 1 was − 12.61 ± 5.83 mm, while in group 2, it was − 11.40 ± 5.14 mm, as shown in Table 1.

The OCT measurements of group 1 and in comparison, with those of the age-matched control group revealed that the central macular thickness was significantly lower in the high myopia group and measured 220.91 ± 27.87 μm and 258.23 ± 17.26 μm, respectively, which was statistically significant (*P* < 0.0001). The differences in parafoveal macular thickness in different quadrants and in the perifoveal macular quadrant thickness were statistically significant (*P* < 0.0001), which indicated that the high myopes in the (4–11 years) age group were much thinner than the age-matched emmetropes were.

**Fig. 1:**
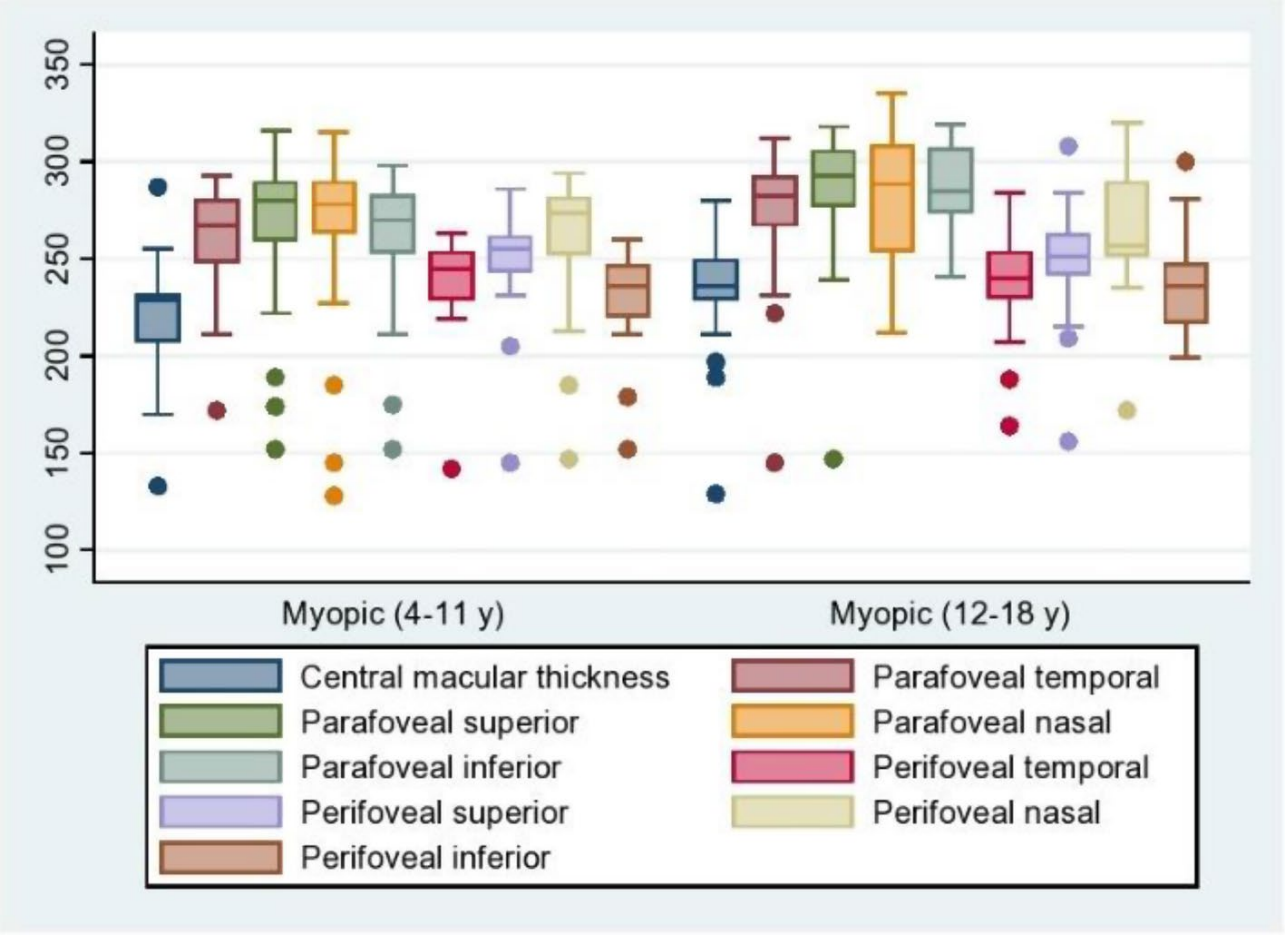
Shows the difference between group 1 (myopic 4–11 years) and group 2 myopic (12–18 years) in the central macular thickness, parafoveal and perifoveal in different sectors

In the older patients with high myopia (group 2), the CMT was 236.32 ± 27.76, and that in the age-matched controls was 247.09 ± 16.81. However, the difference was not statistically significant, *P* = 0.09. The differences in thicknesses of the parafoveal and perifoveal sectors were found to be statistically significant (*P* < 0.0001), (Fig. 1).

**Table 1:** Demographic and refractive data of group 1 (myopic 4–11 years) versus normal controls (4–11 years) and group 2 (myopic 12–18 years) versus normal controls (12–18 years)

Variable	Group 1 Myopic (4–11 years) N = 34	Normal control (4–11 years) N = 34	P value	Variable	Group 2 (Myopic 12–18 years) N = 34	Normal control (12–18 years) N = 34	P value
**Age/years**				**Age/years**			
Mean ± SD Median (range)	7.66 ± 2.24 7 (4:11)	7.76 ± 1.95 8 (4:11)	0.77	Mean ± SD Median (range)	15.46 ± 2.24 16 (12:18)	15.18 ± 1.60 15 (12.5:18)	0.41
**Gender**				**Gender**			
Female Male	24 (70.59%) 10 (29.41%)	17 (50.00%) 17 (50.00%)	0.08	Female Male	21 (61.76%) 13 (38.24%)	19 (55.88%) 15 (44.11%)	0.55
**Eye**				**Eye**			
Left Right	16 (47.06%) 18 (52.94%)	16 (47.06%) 18 (52.94%)	1.00	Left Right	16 (47.06%) 18 (52.94%)	19 (55.88%) 15 (44.11%)	0.39
**Axial length**				**Axial length**			
Mean ± SD	27.82 ± 1.30	23.07 ± 0.90	< 0.0001	Mean ± SD	27.97 ± 0.83	23.05 ± 0.74	< 0.0001
**Manifest refraction**				**Manifest refraction**			
Mean ± SD	-12.61 ± 5.83	0.02 ± 0.54	< 0.0001	Mean ± SD	-11.40 ± 7.40	0 ± 0.42	< 0.0001
**Cycloplegic refraction**				**Cycloplegic refraction**			
Mean ± SD	-11.95 ± 4.70	1.46 ± 0.39	< 0.0001	Mean ± SD	-12.31 ± 3.21	1.30 ± 0.47	< 0.0001
**K1**				**K1**			
Mean ± SD	43.84 ± 2.16	42.29 ± 1.18	0.03	Mean ± SD	43.14 ± 1.44	42.48 ± 1.06	0.006
**K2**				**K2**			
Mean ± SD	44.62 ± 2.03	42.65 ± 0.81	< 0.0001	Mean ± SD	44.68 ± 1.71	42.80 ± 0.77	< 0.0001
**BCVA**				**BCVA**			
Mean ± SD	0.63 ± 0.15	0.95 ± 0.07	< 0.0001	Mean ± SD	0.63 ± 0.15	0.95 ± 0.07	< 0.0001

The macular volume was significantly lower in the high myopia patients in both groups than in the comparable age-matched controls, (*P* < 0.0001) (Table 2).

A comparison of the two groups of high myopia patients revealed that central macular thickness and parafoveal thickness were significantly greater in group 2 than in group 1, (*P* < 0.001); however, regarding perifoveal thickness, the difference between the two groups was not statistically significant (Table 3).

**Fig. 2:**
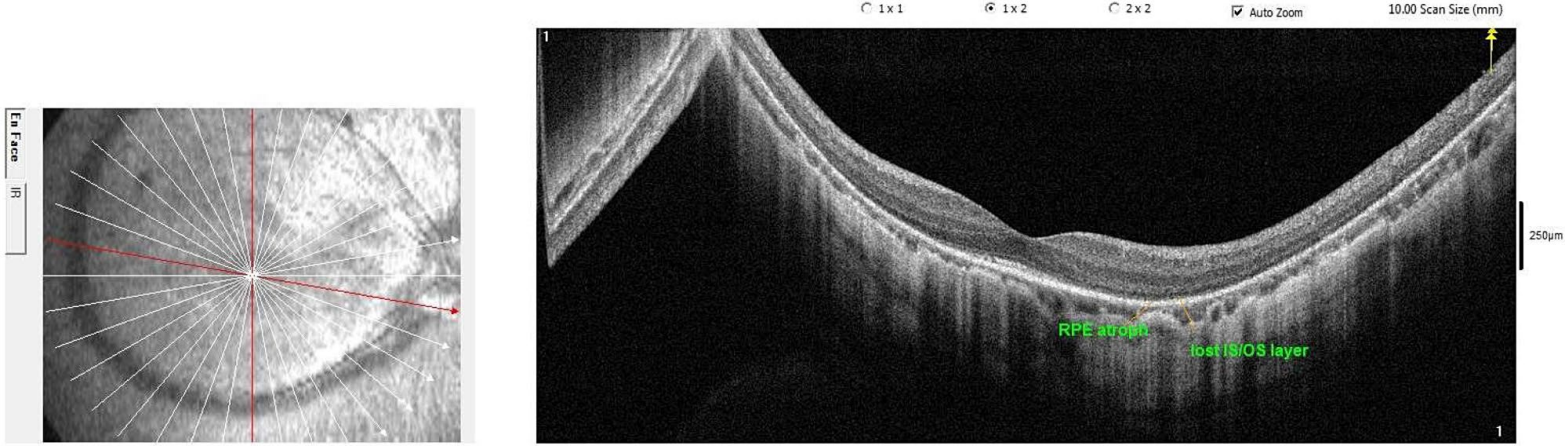
Shows RPE atrophy and loss of photoreceptor (IS/OS) layer in a highly myopic child of fourteen years old with a refraction − 14.50 D and an axial length 28.79 mm

Regarding the structural changes that occurred in the two groups, in group 1 and group 2, no macular holes, no tractional maculopathy, no macular schisis, no lacquer cracks, or no neovascular lesions were detected. However, patchy RPE atrophic changes were observed in 3 eyes (in 8.82% of cases) and 5 eyes (14.70% of cases) in group 1 and group 2, respectively (Fig. 2), (see additional file 4).

**Fig. 3:**
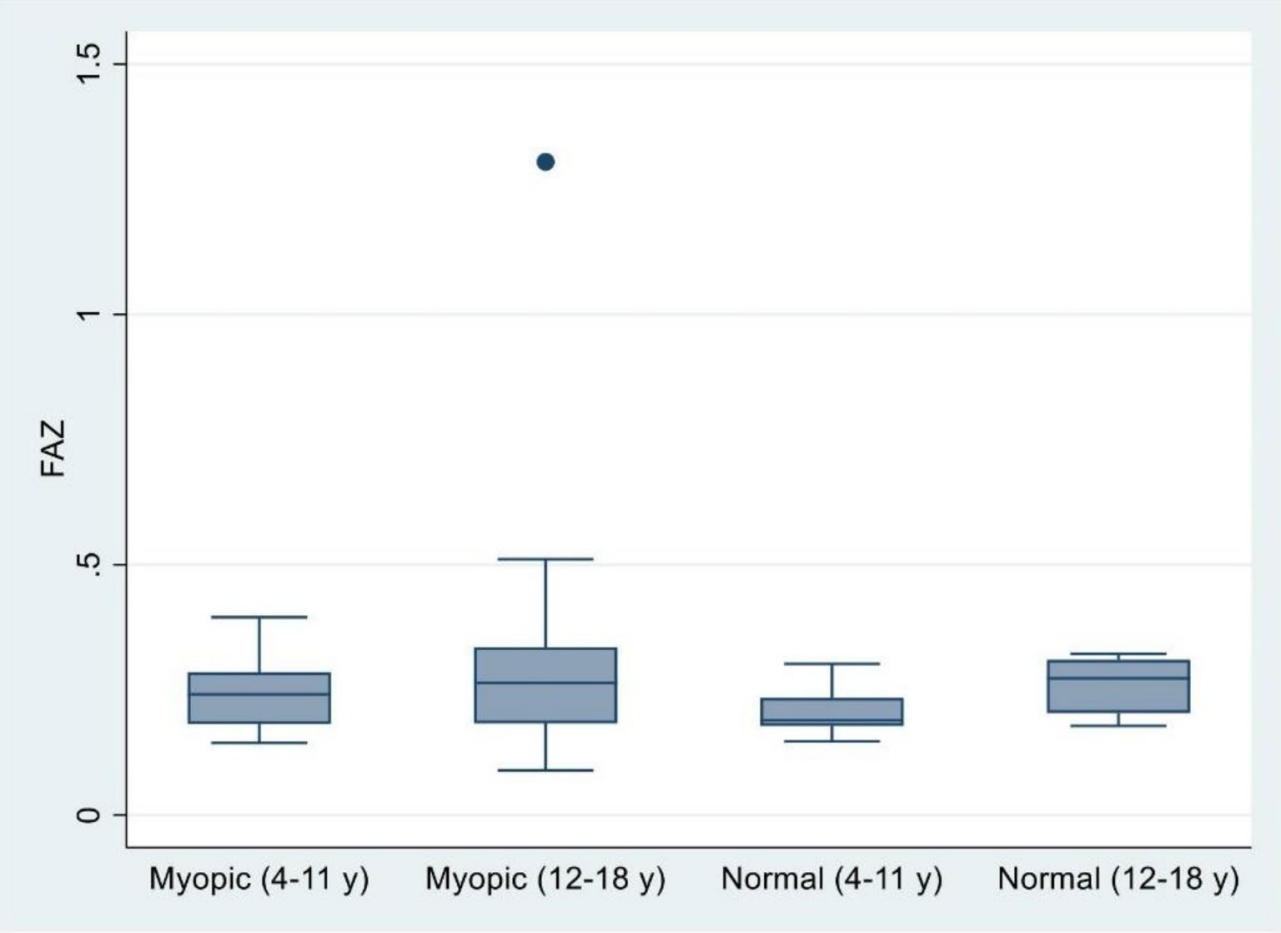
Demonstrates the foveal avascular zone (FAZ) in the myopic groups and their comparable age control groups

According to the OCTA findings of both groups and the comparable age-matched groups of emmetropes, the FAZ was wider in group 1 than in the age-matched emmetropes. There was a statistically significant difference (*P* = 0.02). The FAZ in group 2 was wider than that in the age-matched group, but the difference was not statistically significant (Fig. 3).

According to the comparison between the two high myopia groups, the FAZ seemed to be larger in the older group than in the younger group, but the difference was not statistically significant (Fig. 3).

Analysis of data of the vessel density in the superficial and deep capillary plexuses was performed. Regarding the vessel density (the whole image), it was found that the high myopes in both age groups were significantly lower than that in the age-matched group, for both the deep and superficial capillary plexuses (Tables 4 and 5).

**Table 2:** shows the OCT thickness in group 1 group (4–11 years old) versus age matched controls [4–11] and in group 2 (12–18 years old) versus age matched control (12–18 years)

OCT Parameter	Group 1 Myopes (4–11 years)	Controls 4–11 years)	P value	OCT Parameter	Group 2 Myopes (12–18 years)	Controls 12–18 years)	P value
**Central macular thickness**				**Central macular thickness**			
Mean ± SD	220.91 ± 27.87	258.23 ± 17.26	< 0.0001	Mean ± SD	236.32 ± 27.76	247.09 ± 16.81	0.09
**Parafoveal temporal**				**Parafoveal temporal**			
Mean ± SD	261.06 ± 27.17	298.03 ± 10.41	< 0.0001	Mean ± SD	274.65 ± 32.34	298.42 ± 11.48	0.0001
**Parafoveal superior**				**Parafoveal superior**			
Mean ± SD	270.71 ± 36.83	306.62 ± 6.42	< 0.0001	Mean ± SD	285.65 ± 31.14	305.91 ± 6.34	0.0001
**Parafoveal nasal**				**Parafoveal nasal**			
Mean ± SD	266.97 ± 41.40	311.47 ± 10.92	< 0.0001	Mean ± SD	278.82 ± 33.75	310.09 ± 10.49	< 0.0001
**Parafoveal inferior**				**Parafoveal inferior**			
Mean ± SD	262.62 ± 32.41	302.85 ± 8.23	< 0.0001	Mean ± SD	285.71 ± 23.56	302.58 ± 8.51	0.001
**Perifoveal temporal**				**Perifoveal temporal**			
Mean ± SD	239.41 ± 22.16	266.35 ± 8.42	< 0.0001	Mean ± SD	241.65 ± 25.08	263.48 ± 10.16	< 0.0001
**Perifoveal superior**				**Perifoveal superior**			
Mean ± SD	251.35 ± 25.63	284.29 ± 2.78	< 0.0001	Mean ± SD	250.76 ± 26.62	284.48 ± 2.66	< 0.0001
**Perifoveal**				**Perifoveal**			
**nasal** Mean ± SD	262.79 ± 30.94	295.76 ± 7.75	< 0.0001	**nasal** Mean ± SD	267.15 ± 30.25	296.82 ± 8.99	< 0.0001
**Perifoveal inferior**				**Perifoveal inferior**			
Mean ± SD	232.91 ± 22.01	269.68 ± 8.90	< 0.0001	Mean ± SD	238.71 ± 23.99	272.91 ± 5.44	< 0.0001
**Macular Volume**				**Macular Volume**			
Mean ± SD	6.28 ± 0.52	7.33 ± 0.38	< 0.0001	Mean ± SD	6.41 ± 0.53	7.21 ± 0.24	< 0.0001

The parafoveal and perifoveal vessel densities in the superficial and deep capillary plexuses were significantly lower in the high myopia groups than in the age-matched emmetropes, in most sectors with the exception in group 1: on superficial layer: parafovea total, temporal and superior; on deep layer parafoveal total, nasal and inferior (Table 4), and in group 2: Parafoveal temporal of superficial layer (Table 5).

**Fig. 4:**
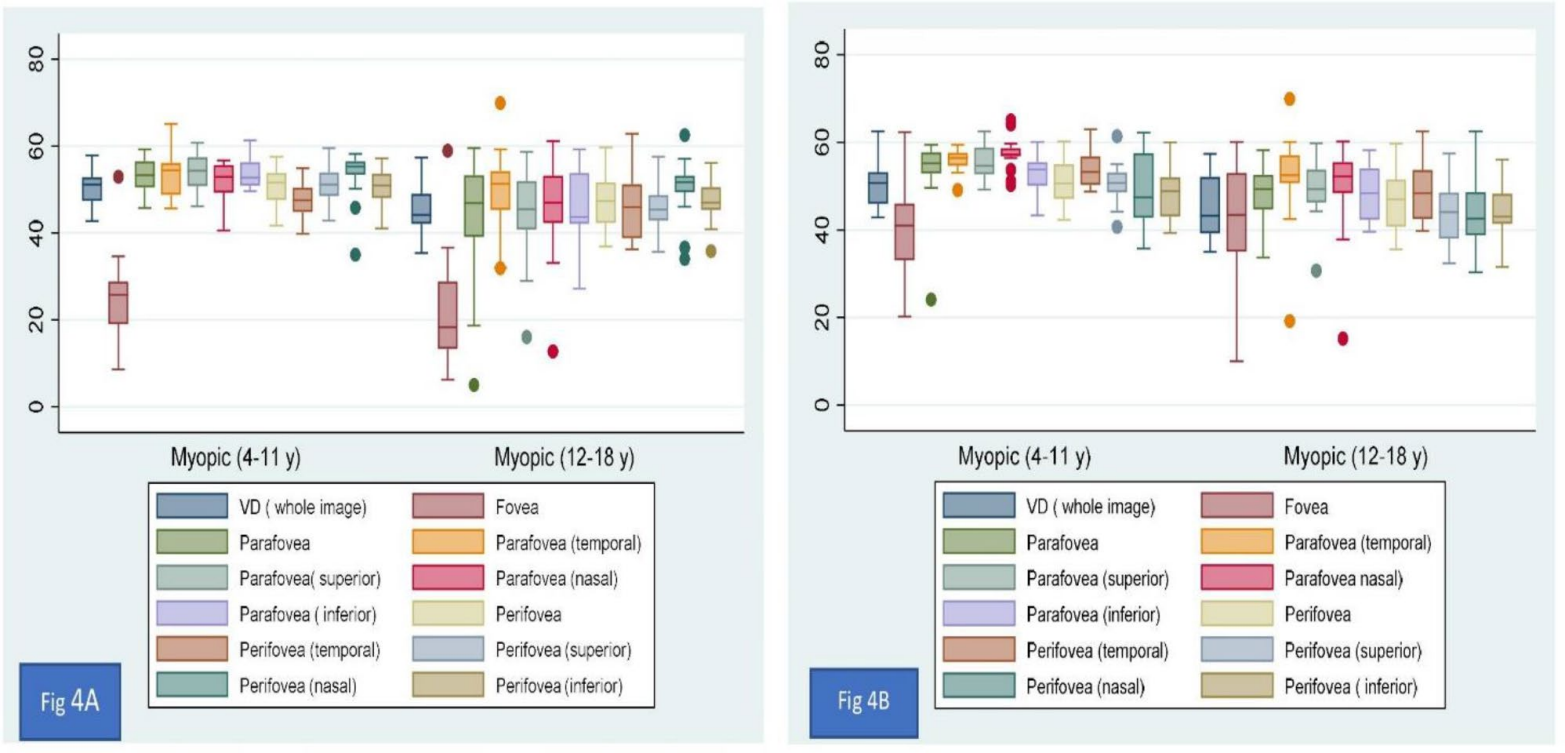
**(A**) & (**B**) shows the superficial vessel density and deep vessel density measured by OCTA

A comparison of vessel density between group 1 and group 2 revealed that the vessel density was lower in older myopic cases than in younger cases, and the difference was evident in all parafoveal and perifoveal sectors and statistically significant except parafoveal temporal and perifoveal temporal sectors in the superficial vessel density (Table 6; Fig. 4A and B).

**Table 3:** Comparison of OCT macular parameters between Group 1 myopic patients (4–11 years) with Group 2 myopic patients (12–18 years)

Variable	Myopic (4–11 years) N = 34	Myopic (12–18 years) N = 34	P value
**Central macular thickness**			
Mean ± SD	220.91 ± 27.87	236.32 ± 27.76	0.004
**Parafoveal temporal**			
Mean ± SD	261.06 ± 27.17	274.65 ± 32.34	0.01
**Parafoveal superior**			
Mean ± SD	270.71 ± 36.83	285.65 ± 31.14	0.03
**Parafoveal nasal**			
Mean ± SD	266.97 ± 41.40	278.82 ± 33.75	0.24
**Parafoveal inferior**			
Mean ± SD	262.62 ± 32.41	285.71 ± 23.56	0.002
**Perifoveal temporal**			
Mean ± SD	239.41 ± 22.16	241.65 ± 25.08	0.98
**Perifoveal superior**			
Mean ± SD	251.35 ± 25.63	250.76 ± 26.62	0.46
**Perifoveal nasal**			
Mean ± SD	262.79 ± 30.94	267.15 ± 30.25	0.94
**Perifoveal inferior**			
Mean ± SD	232.91 ± 22.01	238.71 ± 23.99	0.98
**Macular Volume**			
Mean ± SD	6.28 ± 0.52	6.41 ± 0.53	0.28

**Table 4:** Shows a comparison between group 1 (myopic 4–11 years) with the normal controls (4–11 years) as regard the vessel density at the superficial capillary plexus and deep capillary plexus

Superficial vessel density					Deep vessel density				
Variable	Group 1 Myopic (4–11 years)	Normal control (4–11 years)	P value	Variable		Group 1 myopic (4–11 years)	Normal control (4–11 years)	P value	
**VD (whole image)**				**VD (whole image)**					
Mean ± SD	50.42 ± 3.97	53.48 ± 2.95	0.001	Mean ± SD		50.70 ± 5.49	53.29 ± 2.78	0.02	
**Parafovea**				**Parafovea**					
Mean ± SD	53.23 ± 3.57	53.86 ± 2.84	0.40	Mean ± SD		53.37 ± 55.2	56.43 ± 2.91	0.15	
**Parafoveal temporal**				**Parafoveal temporal**					
Mean ± SD	53.21 ± 4.75	53.55 ± 2.08	0.99	Mean ± SD		56.01 ± 2.53	61.07 ± 3.36	< 0.0001	
**Parafoveal superior**				**Parafoveal superior**					
Mean ± SD	53.87 ± 4.26	54.26 ± 2.34	1.00	Mean ± SD		55.19 ± 3.78	57.1 ± 3.14	0.02	
**Parafoveal nasal**				**Parafoveal nasal**					
Mean ± SD	51.67 ± 4.76	57.62 ± 2.97	< 0.0001	Mean ± SD		57.61 ± 3.85	56.46 ± 2.99	0.22	
**Parafoveal inferior**				**Parafoveal inferior**					
Mean ± SD	54.17 ± 3.67	60.98 ± 3.67	< 0.0001	Mean ± SD		52.78 ± 4.25	53.85 ± 2.97	0.61	
**Perifovea**				**Perifovea**					
Mean ± SD	50.72 ± 3.91	62.33 ± 7.56	< 0.0001	Mean ± SD		50.64 ± 5.18	53.71 ± 3.12	0.02	
**Perifoveal temporal**				**Perifoveal temporal**					
Mean ± SD	47.58 ± 3.87	66.44 ± 6.88 64.95 (53.6:75.53)	< 0.0001	Mean ± SD		54.04 ± 4.36	54.71 ± 2.16	0.15	
**Perifoveal superior**				**Perifoveal superior**					
Mean ± SD	50.97 ± 4.36	70.55 ± 5.37	< 0.0001	Mean ± SD		50.50 ± 5.35	53.28 ± 2.88	0.02	
**Perifoveal nasal**				**Perifoveal nasal**					
Mean ± SD	53.53 ± 5.55	74.81 ± 6.44	< 0.0001	Mean ± SD		49.21 ± 7.80	52.61 ± 2.38	0.03	
**Perifoveal inferior**				**Perifoveal inferior**					
Mean ± SD	50.12 ± 4.22	78.25 ± 7.19	< 0.0001	Mean ± SD		48.18 ± 5.64	52.01 ± 3.25	0.003	

**Table 5:** Comparison of vessel density at the superficial capillary plexus and deep capillary plexus between group 2 (myopic 12–18 years old) with normal controls (12–18 years old)

Superficial vessel density				Deep vessel density			
Variable	**Group 2** **Myopic (12–18 years)**	**Normal control (12–18 years)**	**P value**	**Variable**	**Group 2 Myopic (12–18 years)**	**Normal control (12–18 years)**	**P value**
**VD (whole image)**				**VD (whole image)**			
Mean ± SD	45.8 ± 5.82	53.36 ± 2.71	< 0.0001	Mean ± SD	44.84 ± 6.79	52.76 ± 2.94	< 0.0001
**Parafovea**				**Parafovea**			
Mean ± SD	44.26 ± 14.58	54.62 ± 1.80	0.0001	Mean ± SD	48.78 ± 6.80	56.38 ± 2.58	< 0.0001
**Parafoveal temporal**				**Parafoveal temporal**			
Mean ± SD	49.69 ± 9.42	53.64 ± 1.95	0.02	Mean ± SD	52.04 ± 10.73	60.35 ± 3.26	< 0.0001
**Parafoveal superior**				**Parafoveal superior**			
Mean ± SD	44.11 ± 11.25	54.55 ± 2.50	< 0.0001	Mean ± SD	48.87 ± 7.28	57.26 ± 3.16	< 0.0001
**Parafoveal nasal**				**Parafoveal nasal**			
Mean ± SD	44.34 ± 12.27	57.22 ± 3.41	< 0.0001	Mean ± SD	49.54 ± 10.41	57.14 ± 2.52	< 0.0001
**Parafoveal inferior**				**Parafoveal inferior**			
Mean ± SD	45.52 ± 7.49	60.62 ± 4.07	< 0.0001	Mean ± SD	48.46 ± 6.22	54.19 ± 2.83	0.002
**Perifovea**				**Perifovea**			
Mean ± SD	46.83 ± 5.83	64.05 ± 4.73	< 0.0001	Mean ± SD	45.98 ± 7.21	53.61 ± 3.22	< 0.0001
**Perifoveal temporal**				**Perifoveal temporal**			
Mean ± SD	45.66 ± 7.36	66.60 ± 7.12	< 0.0001	Mean ± SD	48.94 ± 7.08	54.95 ± 2.28	0.0001
**Perifoveal superior**				**Perifoveal superior**			
Mean ± SD	46.05 ± 4.92	70.11 ± 7.52	< 0.0001	Mean ± SD	44.98 ± 7.66	52.49 ± 3.36	< 0.0001
**Perifoveal nasal**				**Perifoveal nasal**			
Mean ± SD	50.24 ± 7.12	73.13 ± 9.08	< 0.0001	Mean ± SD	53.63 ± 8.46	52.09 ± 2.97	< 0.0001
**Perifoveal inferior**				**Perifoveal inferior**			
Mean ± SD Median (range)	47.39 ± 5.04	76.18 ± 10.58	< 0.0001	Mean ± SD	43.61 ± 7.20	52.01 ± 3.55	< 0.0001

**Table 6:** Comparison of the vessel density in superficial and deep capillary plexuses between group 1 (myopia 4–11) and group 2 (myopia12–18)

Superficial vessel density	Myopic (4-11) years)	Myopic (12-18 years)	P value	Depp vessel density	Myopic (4-11 years)	Myopic (12-18 years)	P value
	N=34	N=34			N=34	N=34	
**VD (whole image)**				**VD (whole image)**			
Mean ± SD	50.42±3.97	45.8±5.82	0.001	Mean ± SD	50.70±5.49	44.84±6.79	0.0003
**Parafovea**				**Parafovea**			
Mean ± SD	53.23±3.57	44.26±14.58	0.003	Mean ± SD	53.37±55.2	48.78±6.80	0.0001
**Parafoveal temporal**				**Parafoveal temporal**			
Mean ± SD	53.21±4.75	49.69±9.42	0.06	Mean ± SD	56.01±2.53	52.04±10.73	0.008
**Parafoveal superior**				**Parafoveal superior**			
Mean ± SD	53.87±4.26	44.11±11.25	<0.0001	Mean ± SD	55.19±3.78	48.87±7.28	<0.0001
**Parafoveal nasal**				**Parafoveal nasal**			
Mean ± SD	51.67±4.76	44.34±12.27	0.001	Mean ± SD	57.61±3.85	49.54±10.41	<0.0001
**Parafoveal inferior**				**Parafoveal inferior**			
Mean ± SD	54.17±3.67	45.52±7.49	<0.0001	Mean ± SD	52.78±4.25	48.46±6.22	0.004
**Perifovea**				**Perifovea**			
Mean ± SD	50.72±3.91	46.83±5.83	0.002	Mean ± SD	50.64±5.18	45.98±7.21	0.004
**Perifoveal temporal**				**Perifoveal temporal**			
Mean ± SD	47.58±3.87	45.66±7.36	0.13	Mean ± SD	54.04±4.36	48.94±7.08	0.0003
**Perifoveal superior**				**Perifoveal superior**			
Mean ± SD	50.97±4.36	46.05±4.92	<0.0001	Mean ± SD	50.50±5.35	44.98±7.66	0.002
**Perifoveal nasal**				**Perifoveal nasal**			
Mean ± SD	53.53±5.55	50.24±7.12	0.002	Mean ± SD	49.21±7.80	53.63±8.46	0.008
**Perifoveal inferior**				**Perifoveal inferior**			
Mean ± SD	50.12±4.22	47.39±5.04	0.01	Mean ± SD	48.18±5.64	43.61±7.20	0.01

## Discussion

High myopia results in eye morbidities and pathologies, such as chorioretinal atrophy, myopic maculopathy, glaucoma, and retinal detachment [19]. Pathologic myopia is defined as high myopia with complicated issues in the posterior segment related to excessive and progressive elongation of the globe.

In general, analyzing the macular thickness in the ETDRS areas revealed that the fovea is the thinnest, the inner parafoveal ring is the thickest area, and the outer ring perifoveal area is midway between the fovea and parafoveal thickness [20].

In previous studies, OCT measurements have been shown to be affected by axial length, as longer axial length is associated with thinner OCT measurements in comparison with those shorter axial lengths [21], therefore, the measurements in this study were adjusted using a modified Littmann’s formula that used a magnification factor of the eye to make this correction [15] to avoid bias.

The relationship between central macular thickness and the progression of myopia in children is still controversial, as many studies have reported that CMT is significantly decreased in highly myopic individuals [22, 23], which agrees with our findings. Others have shown that increasing axial myopia is associated with increased CMT and reduced parafoveal and perifoveal retinal thickness [20].

We found that parafoveal and perifoveal thicknesses were lower in highly myopic children than in controls. These findings are consistent with those of Ziylan et al., who studied parafoveal and perifoveal thickness in high myopic children aged 3–7 years and they differ as regard the central macular thickness results. Ziylan also reported that the average macular volume was substantially lower in eyes with high myopia which agreed with our study in this result [23]. This study also agrees with a study on Singaporean high myopic children that showed that macular thickness and macular volume were affected by long axial length. Luo et al. postulated that anatomical change begins early in childhood in high myopia [24].

Chen et al. compared the children of high myopia to those with low myopia. They reported that those with moderate to high myopia had a greater foveal thickness, thinner quadrant-specific thickness in the outer ring (perifoveal), and a smaller average macular thickness/volume [20].

However, other studies performed on high myopia in adulthood reported a positive correlation between axial length and central macular thickness. However, the parafoveal, perifoveal, and macular volumes were much less than those of the comparative controls. These studies explained that relatively increased central macular thickness might be strongly affected or confused by myopic retinoschisis or edema associated with traction [25]. Song et al. came to the same conclusion [26]. Additionally, Zhao reported the same results that parafoveal and perifoveal sectors were more affected in patients with high myopia and presented with significant thinning. The results of this study and those of previous studies correlated with the idea that posterior stretching in high myopia leads to retinal thinning [27]. Wang reported that the retinal thickness decreased with increasing axial length in patients with high myopia and the thickness of individual retinal layers decreased [28]. According to Salehi et al., retinal thickness in the group with high myopia became less than that of the low myopia group in the parafoveal and perifoveal areas [29].

In a comparison between the two groups of high myopia, revealed that the central macular thickness was significantly greater in adolescents than in children, and most parafoveal macular sector thicknesses were greater in adolescents. These results led us to hypothesize that the central macula tends be thicker with age in high myopia patients as a compensatory mechanism.

This idea agrees with the conclusion postulated before by Wakitani et al. that in myopic eyes central macular thickness increases as a compensatory mechanism at the expense of a thinner peripheral retina to preserve the fovea to maintain the central vision [30]. And this may also explain why many studies have reported thicker central macular thickness in adults with high myopia comparable to age matched emmetropes [16].

In the present study, regarding the vessel density in the superficial capillary plexus in highly myopic children (4–11 years old), there was a statistically significant difference in the whole image. In the parafoveal sectors, the vessel density was decreased in myopes than comparable age-matched controls and the difference is statistically significant in parafoveal nasal and parafoveal inferior sectors. Nonetheless, in most perifoveal sectors, the vessel density was much lower than that of the controls. This finding agrees with that of a previous study by Gotebiewska, who revealed that foveal and parafoveal superficial vessel density is decreased in myopic Caucasian children [31]. In contrast, the vessel density percentage in deep capillary plexuses it was found that the perifoveal sectors in high myopes were much decreased than controls, and the difference is more apparent in perifoveal areas than parafoveal sectors.

The vessel density in deep and superficial plexuses was much lower in the adolescent group with high myopia than in the child group according to the whole images and the parafoveal and perifoveal sectors, and the difference was highly statistically significant.

These findings are consistent with the hypothesis that high myopia with aging results in ischemic changes resulting from a decrease in vessel density in retinal layers. A comparison of the two groups of high myopia revealed statistically significant differences (Fig. 4).

Many studies have reported that vascular changes occur in high myopia. These changes could be associated with the pathogenesis of myopia. For instance, Shimada et al. concluded that retinal blood flow was lower in patients with high myopia, primarily because of a narrowing retinal vessel diameter. This relative ischemia may play a role in chorioretinal atrophy [32]. Al-sheikh et al. reported that the retinal capillary microvasculature density decreased, and the flow deficit region was higher in highly myopic eyes [33], FAN et al. also suggested that with increasing myopia, the vascular density decreased in the macular region [34].

The FAZ is a highly specialized area for accurate vision [35]. Changes in FAZ configuration and size are important in prognosis of illnesses and are related to vision conditions [36]. However, no normative data for children and adolescents of any age have yet been obtained. Most of the studies have studied pediatric groups with various retinal diseases in comparison to healthy control groups, as in sickle cell retinopathy [37], as the FAZ was larger. However, Araki reported that the FAZ was smaller in eyes with amblyopia than in fellow eyes [38]. In this study, the FAZ was larger in high myopic kids and high myopic adolescents, which agreed with the findings of Chen et al., who found that high myopia patients had a larger FAZ than did emmetropes [39]. Wang evaluated retinal vascular density and FAZ in youth with high myopia and reported that axial length was negatively correlated with deep and superficial macular vascular density and that the FAZ was larger in high myopia [40]. Wong reported that vessel density was lower in adolescents with high myopia [41].

No CNVs were reported in either group of high myopia patients. This result agrees with the lack of reports of CNV due to myopia [42]. The results showed no reported posterior staphyloma, traction maculopathy, or macular holes in either myopic group. However, patchy RPE atrophy in the macula was detected in both groups, and the percentage of patients with RPE atrophy increased in the adolescent group (group 2). These findings agree with previous studies showing that RPE atrophic changes occur in a patchy or diffuse manner appeared in high myopia and increased with age [42].

RPE atrophic changes could be present in the form of rounded or irregular changes. It was postulated that excessive axial elongation could activate stress in the posterior pole with chronic ischemia, which could cause local or diffuse deterioration of the RPE and/or choroid. Additionally, any progressive change can trigger pathological changes in other tissues and induce CNV development [43].

In this study, the RPE atrophic changes were patchy in children and adolescents. With aging and further axial elongation, the atrophic changes may enlarge, become more diffuse, and affect both the sclera and choroid.

To our knowledge, this study is one of the earliest investigations describing the quantitative microvascular characteristics of the retina in high axial myopia in children and adolescents by OCTA. Furthermore, it included an age-matched control group. Although we examined a larger population, the low signal measurements on OCTA and motion artifacts at this young age led to the exclusion of many participants’ measurements and data. However, Further studies are needed to follow the microvascular changes that may occur with aging in the same participants in a longitudinal manner.

### Electronic supplementary material

Below is the link to the electronic supplementary material.

**Supplementary Material 1:** Figure legends

**Supplementary Material 2:** Demonstrates the OCTA measurements the vessel density in the superficial capillary plexus automatically by inner software

**Supplementary Material 3:** An image demonstrates Measurements of the macular analysis included central macular thickness (central 1 mm disc) and macular thickness in two concentric circles of 3 mm (parafoveal circle) and 6 mm (perifoveal circle) diameters correspondingly centered at the fovea in a highly myopic boy of 8 years old

**Supplementary Material 4:** An image demonstrates FAZ measurement which was measured automatically by OCTA inner software, this image was measured in a highly myopic child of 6 years old

**Supplementary Material 5:** An image showing patchy RPE atrophy and photoreceptor disruption in a highly myopic child of 9 years old

## Data Availability

The datasets used and/or analyzed during the current study are available from the corresponding author on reasonable request.

## Abbreviations

RNFL: Retinal Nerve Fiber Layer
OCT: Optical Coherence Tomography
FA: Fluorescein Angiography
OCTA: Optical Coherence Tomography Angiography
m CNV: Myopic choroidal neovascularization
BCVA: Best corrected visual Acuity
ILM: Internal Limiting Membrane
RPE: Retinal Pigment Epithelium
IPL: Inner plexiform Layer
ETDRS: Early Treatment Diabetic Retinopathy Study design
FAZ: Foveal Avascular Zone
Als: axial length of the subject in mm
Alm: axial length assumed for the model eye
CMT: Central Macular Thickness
