# Supplementary material for: Optical coherence tomography angiography of the macula of high myopia in children and adolescents
Source: Int J Retina Vitreous. 2024 Feb 5;10:17. doi: 10.1186/s40942-024-00532-w (PMC10845789; doi:10.1186/s40942-024-00532-w)
Supplement: Supplementary file 1 — Supplementary Material 1: Figure legends [file 40942_2024_532_MOESM1_ESM.docx]

Additional file 1.

Demonstrates the OCTA measurements the vessel density in the superficial capillary plexus automatically by inner software.

Additional file 2.

An image demonstrates Measurements of the macular analysis included central macular thickness (central 1 mm disc) and macular thickness in two concentric circles of 3 mm (parafoveal circle) and 6 mm (perifoveal circle) diameters correspondingly centered at the fovea in a highly myopic boy of 8 years old.

Additional file 3.

An image demonstrates FAZ measurement which was measured automatically by OCTA inner software, this image was measured in a highly myopic child of 6 years old.

Additional file 4.

An image showing patchy RPE atrophy and photoreceptor disruption in a highly myopic child of 9 years old.
