# Supplementary figures and images for: Optical coherence tomography angiography of the macula of high myopia in children and adolescents
Source: Int J Retina Vitreous. 2024 Feb 5;10:17. doi: 10.1186/s40942-024-00532-w (PMC10845789; doi:10.1186/s40942-024-00532-w)

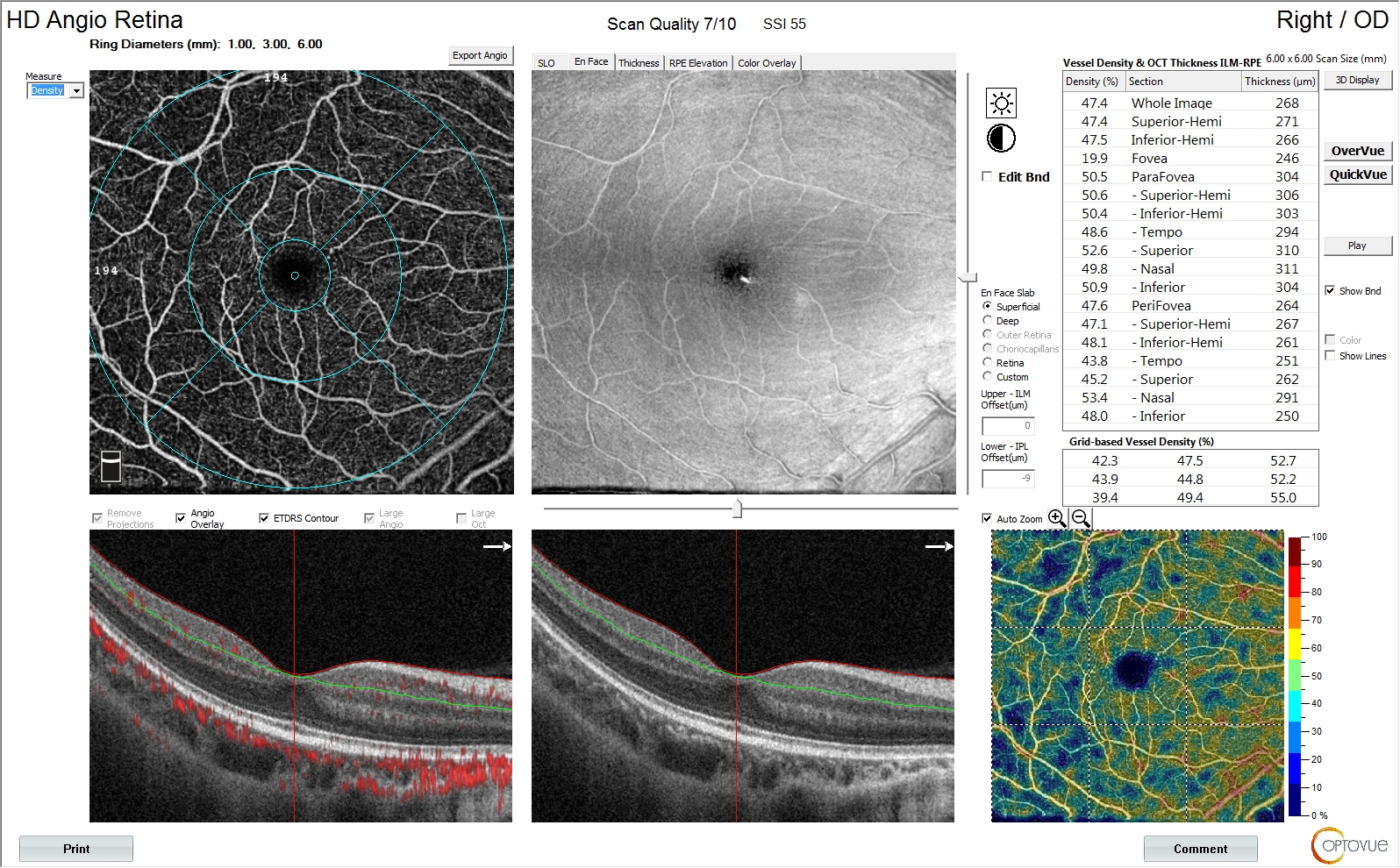

Supplement: Supplementary file 2 — Supplementary Material 2: Demonstrates the OCTA measurements the vessel density in the superficial capillary plexus automatically by inner software [file 40942_2024_532_MOESM2_ESM.jpg]

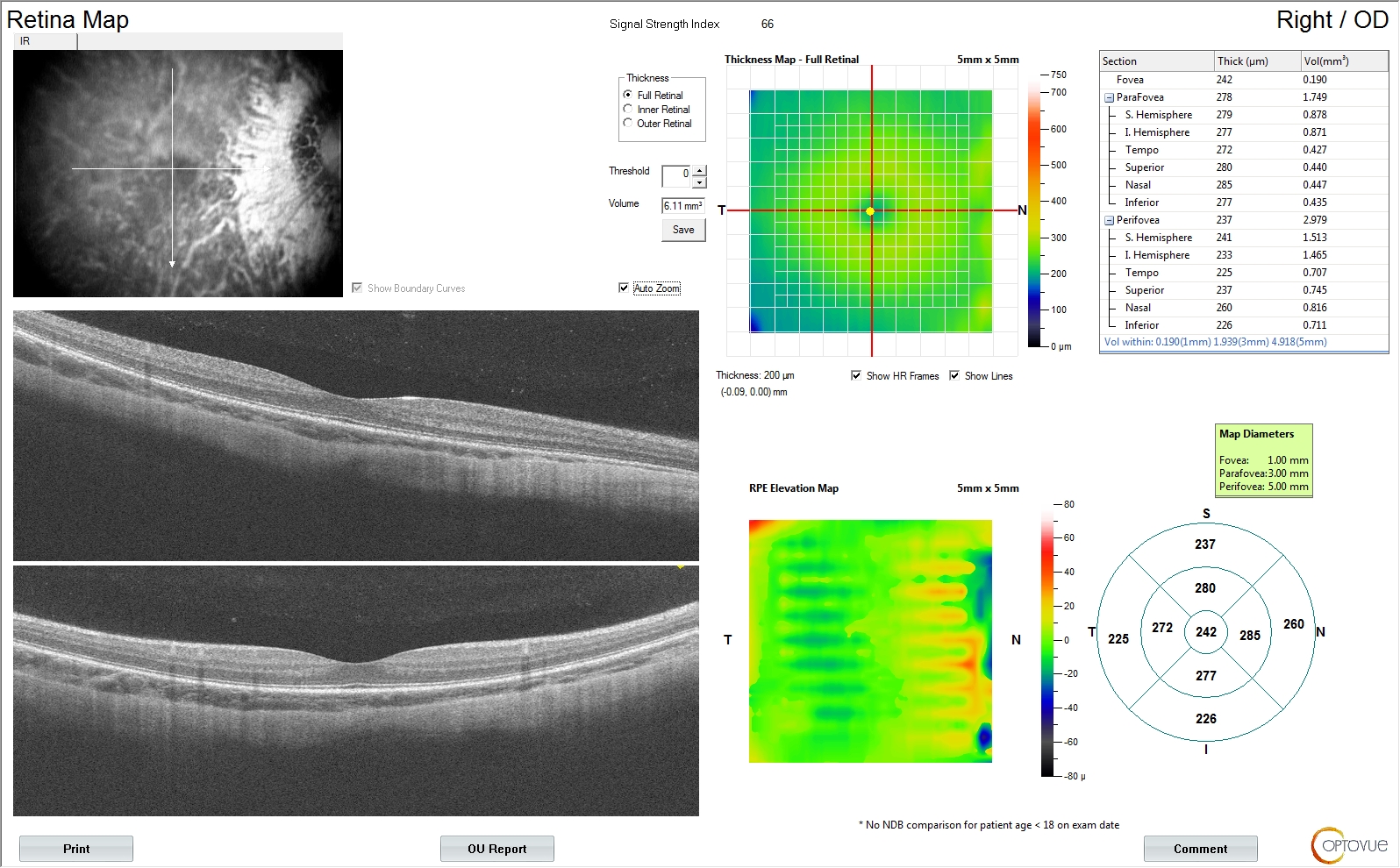

Supplement: Supplementary file 3 — Supplementary Material 3: An image demonstrates Measurements of the macular analysis included central macular thickness (central 1 mm disc) and macular thickness in two concentric circles of 3 mm (parafoveal circle) and 6 mm (perifoveal circle) diameters correspondingly centered at the fovea in a highly myopic boy of 8 years old [file 40942_2024_532_MOESM3_ESM.jpg]

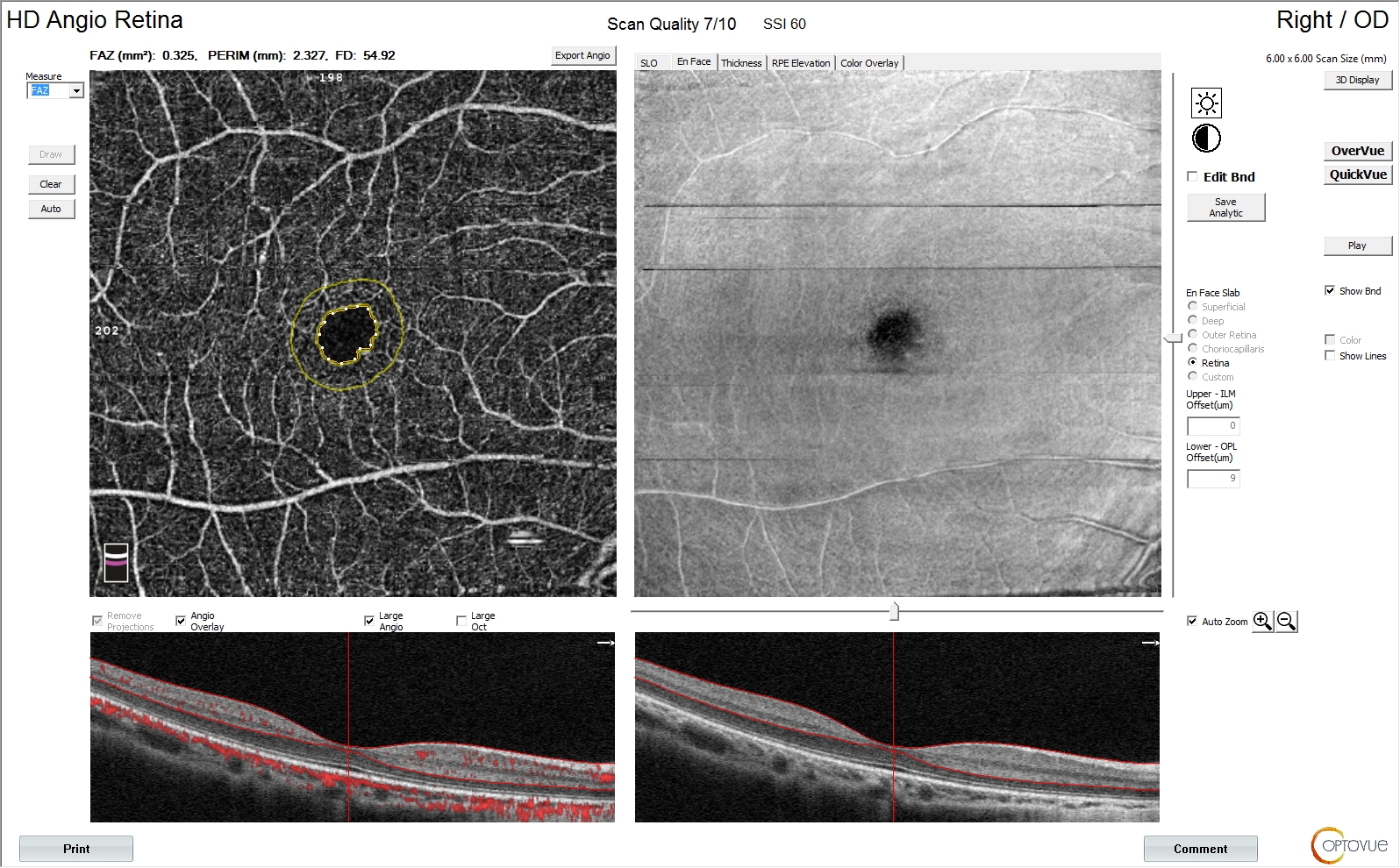

Supplement: Supplementary file 4 — Supplementary Material 4: An image demonstrates FAZ measurement which was measured automatically by OCTA inner software, this image was measured in a highly myopic child of 6 years old [file 40942_2024_532_MOESM4_ESM.jpg]

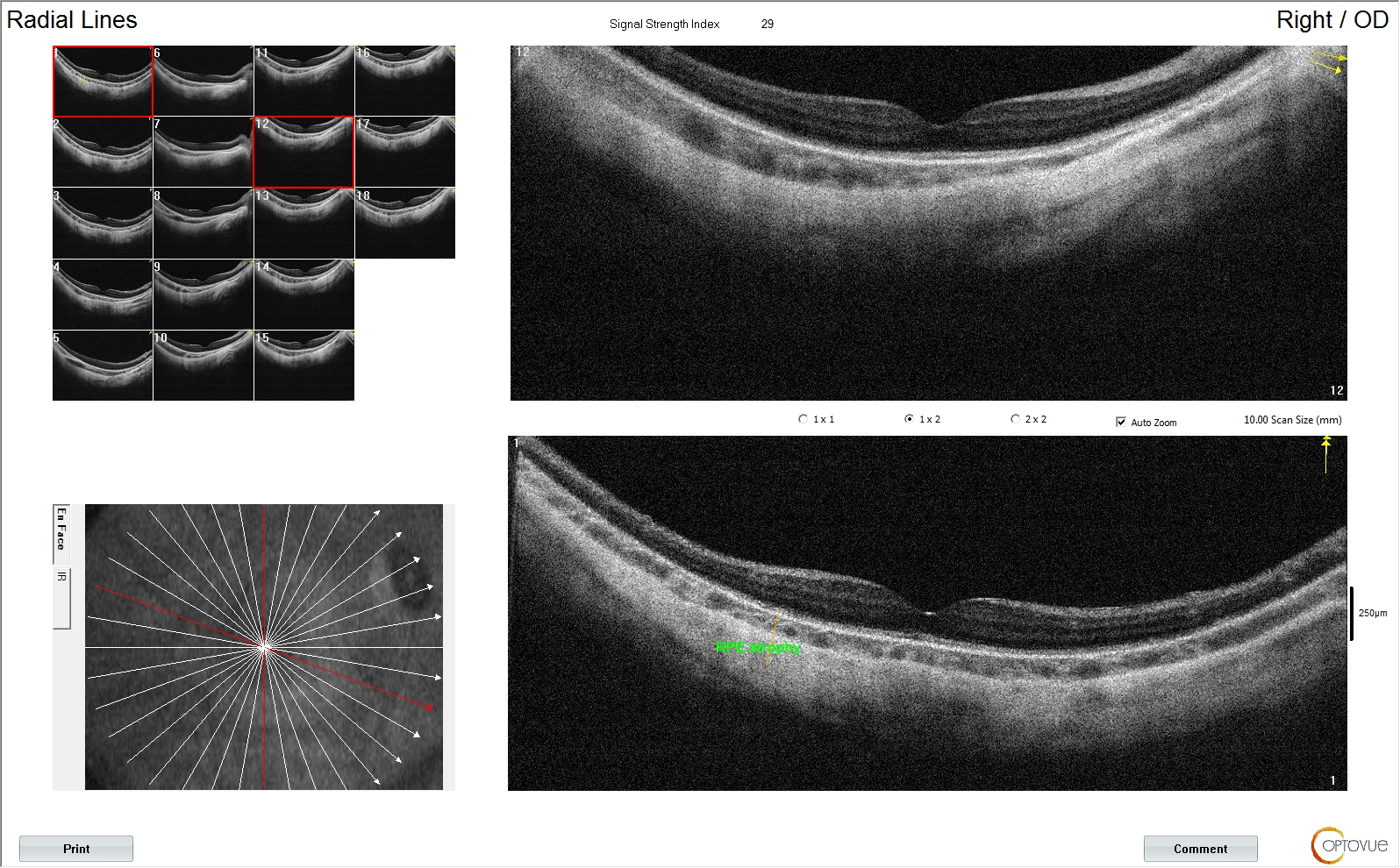

Supplement: Supplementary file 5 — Supplementary Material 5: An image showing patchy RPE atrophy and photoreceptor disruption in a highly myopic child of 9 years old [file 40942_2024_532_MOESM5_ESM.jpg]
